# Plasma Levels of Homocysteine and Cysteine Increased in Pediatric NAFLD and Strongly Correlated with Severity of Liver Damage

**DOI:** 10.3390/ijms151121202

**Published:** 2014-11-17

**Authors:** Anna Pastore, Anna Alisi, Gianna di Giovamberardino, Annalisa Crudele, Sara Ceccarelli, Nadia Panera, Carlo Dionisi-Vici, Valerio Nobili

**Affiliations:** 1 Metabolomics and Proteomics Unit, “Bambino Gesù” Children’s Hospital, IRCCS, Rome 00165, Italy; E-Mails: gianna.digiovamberardino@opbg.net (G.G.); carlo.dionisivici@opbg.net (C.D.-V.); 2 Liver Research Unit, “Bambino Gesù” Children’s Hospital, IRCCS, Rome 00165, Italy; E-Mails: annalisa.crudele@gmail.com (A.C.); sara.ceccarelli@opbg.net (S.C.); nadia.panera@opbg.net (N.P.); valerio.nobili@opbg.net (V.N.); 3 Hepato-Metabolic Disease Unit, “Bambino Gesù” Children’s Hospital, IRCCS, Rome 00165, Italy

**Keywords:** homocysteine, cysteine, cysteinylglycine, glutathione, non-alcoholic fatty liver disease (NAFLD), non-alcoholic steatohepatitis (NASH)

## Abstract

Non-alcoholic fatty liver disease (NAFLD) is a spectrum of metabolic abnormalities ranging from simple triglyceride accumulation in the hepatocytes to hepatic steatosis with inflammation, ballooning and fibrosis. It has been demonstrated that the pathogenesis of NAFLD involves increased oxidative stress, with consumption of the major cellular antioxidant, glutathione (GSH). Liver has a fundamental role in sulfur compound metabolism, although the data reported on plasma thiols status in NAFLD are conflicting. We recruited 63 NAFLD patients, and we analyzed all plasma thiols, such as homocysteine (Hcy), cysteine (Cys), cysteinylglycine (CysGly) and GSH, by high-performance liquid chromatography (HPLC) with fluorescence detection. Hcy, Cys and CysGly plasma levels increased in NAFLD patients (*p* < 0.0001); whereas GSH levels were decreased in NAFLD patients when compared to controls (*p* < 0.0001). On the contrary, patients with steatohepatitis exhibited lower levels of Hcy and Cys than subjects without. Furthermore, a positive correlation was found between Hcy and Cys and the presence of fibrosis in children with NAFLD. Taken together, these data demonstrated a defective hepatic sulfur metabolism in children with NAFLD, and that high levels of Hcy and Cys probably correlates with a pattern of more severe histological liver damage, due to mechanisms that require further studies.

## 1. Introduction

Non-alcoholic fatty liver disease (NAFLD) is a spectrum of hepatic conditions ranging from the most common benign form of simple steatosis to the rare severe form of non-alcoholic steatohepatitis (NASH) [1]. Currently, NAFLD is considered the most common cause of chronic liver disease in adults and children from most of Westernized countries [2,3]. NAFLD prevalence escalation, in these geographical areas is explicable by the strong correlation of this hepatic disease with the obesity and other features of metabolic syndrome [4]. NASH in children is characterized by a specific histological pattern of liver features including not only steatosis, but also portal and lobular inflammation, ballooning degeneration and eventually fibrosis [5]. On the basis of *in vivo* and *in vitro* experimental models, it has been established that NAFLD pathogenesis is multifactorial and involves alterations in pathways regulating the hepatic lipid metabolism and insulin signaling that lead to steatosis, and liver-resident cell reaction to oxidative stress and inflammatory response that promote progression to NASH [6,7]. However, as NAFLD-associated hepatic damage often occurs in the presence of co-morbidities, in a context of metabolic syndrome it is not only limited to liver but also involves other organs including gut and adipose-tissue, which secrete specific mediators (*i.e.*, growth factors and adipokines) contributing to NASH progression [8]. In this picture a steatotic liver pattern displays an increased production of reactive oxygen species (ROS) and oxidative stress inducing changes in mitochondrial function, depletion of ATP, DNA damage, lipid peroxidation, release of cytokines and consequently hepatic inflammation and fibrosis [9,10].

**Figure 1:**
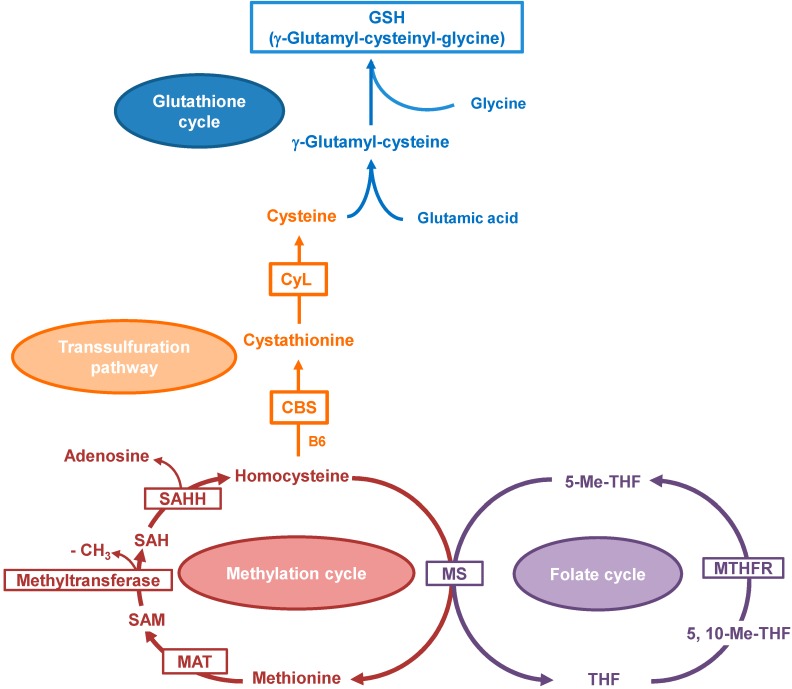
Schematic representation of homocysteine metabolism and intersection with glutathione (GSH) production and folate cycle. Methionine derived from protein degradation is converted into homocysteine by the methylation cycle. Homocysteine is then converted into cysteine by the transsulfuration pathway, providing the substrate for GSH synthesis. The synthesis and catabolism of GSH occurs by a regulated series of enzymatic and membrane transport steps that are collectively referred to as the glutathione cycle. Folate (THF) cycle is also reported.

Homocysteine (Hcy), formed as an intermediary in hepatic methionine metabolism, is a sulfur-containing amino acid. It may either be remethylated to methionine or catabolized in the transsulfuration pathway to cysteine (Cys). Methionine cycle is central to liver one-carbon (C1) metabolism because it maintains methionine homeostasis by generating *S*-adenosylmethionine (SAM) which is the methyl donor in hundreds of methylation reactions including those on RNA, DNA, proteins and lipids. During transmethylation reactions, SAM is reversibly hydrolyzed by *S*-adenosylhomocysteine hydrolase (SAHH) into adenosine and Hcy. Next, Hcy may be remethylated into methionine by methionine synthase (MS) with 5-methyltetrahydrofolate (5-MeTHF) as a methyl donor, or converted into Cys via transsulfuration pathway mediated by the enzymes cystathionine β-synthase (CBS) and cystathionine γ-lyase (CyL). In the folate (THF) cycle 5-MeTHF is generated from 5,10-methyltetrahydrofolate (5,10-MeTHF) by the flavin adenine dinucleotide (FAD)-dependent methylenetetrahydrofolate reductase (MTHFR) [11]. Cys cargo in the portal blood is controlled by glutathione (GSH) synthesis that occurs in the liver. The hepatic GSH provides a continuous source of Cys to support normal metabolism and maintains Cys levels below the threshold of toxicity. Furthermore, it controls redox status in the cell and the essential thiol status of proteins (Figure 1) [12]. In fact, plasma GSH levels are kept relatively low by prior degradation to Cysteinylglycine (CysGly), catalyzed by the cell surface enzyme γ-glutamyl transpeptidase, and next by conversion to cysteine.

In most cases, disturbances in Hcy metabolism and genetically determined defects of enzymes integral to this process are consistent with a condition of abnormally elevated levels of serum Hcy defined as hyperhomocysteinemia, which in turn has been often associated with oxidative stress, type 2 diabetes (T2D), cardiovascular diseases (CVD) and atherosclerosis [13,14]. In addition, other studies reported an association of elevated levels of Hcy and Cys with CVD or T2D [15,16]. In the same diseases, plasma GSH status seems to play a protective role by scavenging free radicals and decreasing the intracellular oxidative stress [17,18].

The liver is central for the synthesis and metabolism of Hcy and related thiols, given that the majority of dietary methionine is metabolized in this organ [19]. Changes in Hcy metabolism were reported during liver damage associated to alterations of lipid metabolism [20,21]. Interestingly, several recent lines of evidence demonstrate that Hcy levels and C1-metabolism may be altered also in experimental models of NAFLD [22,23]. The data reported on plasma levels of Hcy and their potential association with liver damage in adults with NAFLD are conflicting and studies in children affected by the same disease are still lacking [24,25]. However, Kalhan *et al.* in their analysis of plasma metabolomic profiles found that obese subjects with NAFLD had higher plasma concentration of Hcy and total Cys, and lower plasma concentrations of total glutathione than age and sex-matched controls [26]. Furthermore, we previously demonstrated an impairment of GSH metabolism in blood of children with NASH [27].

In the present study, in order to investigate the potential correlation between hyperhomocysteinemia, Hcy-related thiols and the histological pattern that characterizes pediatric NAFLD, we have analyzed the changes of plasma levels of total Hcy, Cys, CysGly and the GSH cycle metabolites in children with biopsy-proven disease.

## 2. Results and Discussion

### 2.1. Results

**Figure 2:**
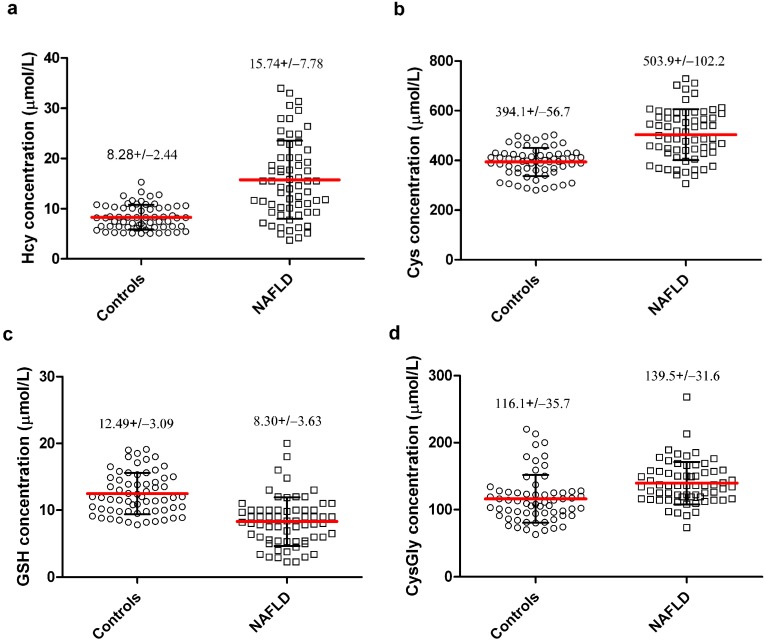
Scatter dot plots of plasma levels of (**a**) homocysteine (Hcy); (**b**) cysteine (Cys); (**c**) GSH and (**d**) Cysteinylglycine (CysGly) in healthy children (Controls) and in age-matched subjects with non-alcoholic fatty liver disease (NAFLD). Mean value (red lines) ± standard deviation (SD, black error bars) are reported. *p* value is <0.0001 for NAFLD *vs.* Controls in all plasma thiols.

Levels of plasma thiols were analyzed in 64 children with biopsy-proven NAFLD and compared with those of the same numbers of age-matched healthy controls. The mean age was 9.1 ± 1.1 years (SD) for the control group and 8.3 ± 3.6 years for the NAFLD group. The male/female ratio was 39:25 in controls and 43:21 in NAFLD subjects. The BMI was similar in the two groups (25.1 ± 2.2 kg/m^2^ for controls and 24.5 ± 3.9 kg/m^2^ for NAFLD). The results of plasma thiols analyses are reported in Figure 2. Plasma levels of Hcy, Cys and CysGly were significantly increased (*p* < 0.0001) in NAFLD children as compared to the control subjects (Figure 2, panels a,b,d). On the contrary the circulating levels of total GSH were significantly lower (*p* < 0.0001) in patients with NAFLD when compared to the control (Figure 2, panel c).

**Figure 3:**
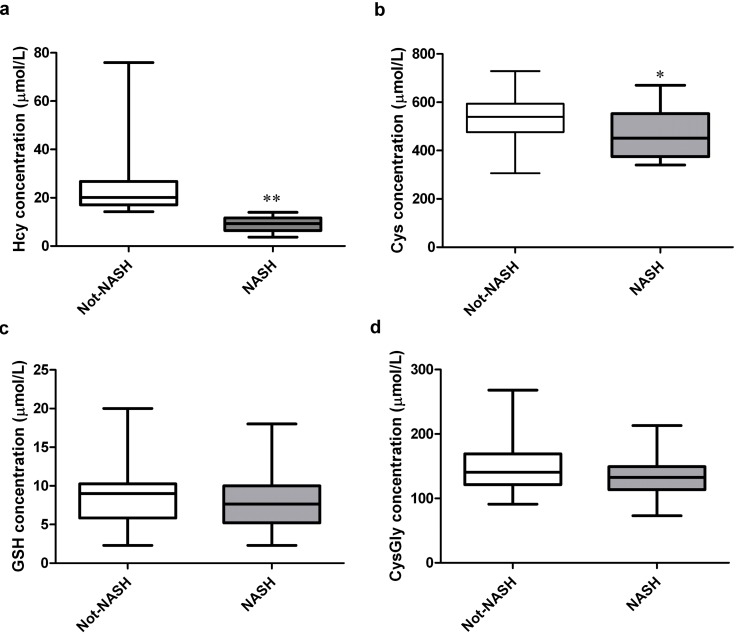
Box plots of plasma levels of (**a**) Hcy; (**b**) Cys; (**c**) GSH and (**d**) CysGly in Not-NASH and NASH children. Mean value ± SD (black error bars) are reported.* *p* < 0.05 and ** *p* < 0.01.

After liver biopsy, the histopathological evaluation confirmed the diagnosis of NASH in 30 and diagnosis of Not-NASH in 34 out of total 64 children with NAFLD. Therefore for further analyses the subjects were divided in two groups according to the severity of disease. As reported in Table 1 the analysis of anthropometrical and metabolic parameters reveals no statistically significant differences between NASH and Not-NASH group except for age, height and weight (*p* < 0.05). Interestingly, when we analyzed the circulating thiols, we observed a significant decrease of Hcy (Figure 3, panel a) and Cys plasma levels (Figure 3, panel b) in NASH compared to Not-NASH children; whereas no statistically significant differences of GSH and CysGly plasma levels (Figure 3, panels c,d) were found between the two groups of patients. The further comparison of values obtained in NASH group *vs.* control group demonstrated that there are no differences in the levels of Hcy, while circulating Cys, GSH and CysGly followed the same trend observed in NAFLD subjects *vs.* controls (data not shown).

As in all NAFLD patients, severity of liver damage has been evaluated by histology (Table 2), the possible correlation between the levels of plasma thiols and the hepatic features that characterize the disease was next analyzed. The plasma levels of Hcy were significantly correlated with those of Cys (Spearman’s *r* = 0.327, *p* = 0.008) and CysGly (Spearman’s *r* = 0.449, *p* = 0.013), and with inflammation (Spearman’s *r* = 0.345, *p* = 0.005) and fibrosis (Spearman’s *r* = 0.449, *p* < 0.001). Similarly, circulating levels of Cys displayed a positive correlation with CysGly (Spearman’s *r* = 0.306, *p* < 0.001) and inflammation (Spearman’s *r* = 0.381, *p* = 0.001) but not with fibrosis (Spearman’s *r* = 0.238, *p* = 0.057). Plasma levels of total GSH had a positive correlation only with CysGly (Spearman’s *r* = 0.476, *p* < 0.001), of which circulating levels were not correlated with histological features of NAFLD. Finally, we performed logistic regression analysis adjusted for age and sex demonstrating that plasma levels of Hcy was significantly associated with fibrosis and the addition of the other variables, including Cys, GSH and CysGly, did not essentially influence the estimates (Table 3).

**Table 1:** Anthropometrics and metabolic parameters in Not-NASH *vs.* non-alcoholic steatohepatitis (NASH) patients.

Parameters	Not-NASH (*n* = 34; 23 Male, 11 Female)	NASH (*n* = 30; 20 Male, 10 Female)	p *
Age (years)	8.4 ± 2.4	9.9 ± 2.3	0.012
Height (cm)	131.2 ± 15.9	141.2 ± 18.6	0.023
Weight (kg)	42.3 ± 14.1	51.8 ± 16.9	0.017
WC (cm)	78.7 ± 10.2	82.1 ± 10.5	0.188
BMI (kg/m^2^)	23.9 ± 4.1	25.1 ± 3.7	0.263
ALT (U/L)	75.7 ± 53.6	83.1 ± 46.4	0.555
AST (U/L)	51.1 ± 19.7	53.2 ±24.8	0.712
γGT (U/L)	24.3 ±14.1	29.1 ± 20.1	0.283
Triglycerides (mg/dL)	100 ± 72	112 ± 79	0.557
Cholesterol (mg/dL)	149 ± 37	151 ± 33	0.821
Glucose (mg/dL)	80 ± 13	83 ± 14	0.437
Insulin (mcU/mL)	11.7 ± 8.4	13.8 ± 7.9	0.432
HOMA	2.4 ± 1.5	2.8 ± 1.4	0.546

Values are mean ± standard deviation; * Student’s *t* test; WC, waist circumference; BMI, body mass index; ALT, alanine aminotransferase; AST, aspartate aminotransferase; γGT, γ-glutamyl-transferase; HOMA, homeostatic model assessment.

**Table 2:** Histological features of children with NAFLD.

Histology	*n*	%
Steatosis		
1	13	20.3
2	39	60.9
3	12	18.8
Inflammation		
0	6	9.4
1	39	60.9
2	18	28.1
Ballooning		
0	28	43.7
1	24	37.5
2	12	18.8
Fibrosis		
0	23	35.9
1	29	45.3
2	7	10.9
3	5	7.9

**Table 3:** Odds ratios for having fibrosis and plasma thiols adjusted for age and sex.

Model	OR	CI	*p*
*1 (Hcy)*	1.141	1.040−1.251	0.005
2 (+ Cys)	1.124	1.022−1.235	0.016
3 (+ Cys + GSH)	1.126	1.024−1.240	0.016
4 (+ Cys + GSH + CysGly)	1.126	1.024−1.240	0.016

### 2.2. Discussion

In the present study we found that children with biopsy proven NAFLD had higher plasma levels of Hcy and Cys and lower levels of circulating total GSH than those observed in age-matched children with no liver disease. These results are consistent with previous studies performed in adult cohorts [26,28]. Indeed, Kalhan *et al.* found that NAFLD subject had increased plasma concentration of Hcy and Cys, and decreased plasma concentrations of total GSH [26]. In addition, the same authors, as well as observed by others, found that hyperhomocysteinemia occurred more in subjects with NASH than in those with simple steatosis [24,26]. Contrarily to these results, but in accordance with a previous study conducted in 39 patients with biopsy-proven NAFLD (54 ± 11 years) compared to 22 healthy controls (52 ± 9 years), we found that children with NASH had lower levels of serum Hcy when compared to Not-NASH subjects [25]. Polyzos *et al.* hypothesized that the downsize of Hcy circulating levels associated to the more severe form of disease could be dependent on a feedback effects to restore adequate levels of antioxidant GSH. This idea could be conceivable in view of Hcy depletion by a strong induction of transsulfuration pathway with a consequent bioavailability of Cys to synthesize GSH. Accordingly to this hypothesis we found a decrease of circulating Cys in NASH children compared to Not-NASH, but in opposite we observed that GSH plasma levels as those of CysGly remained unchanged with respect to disease progression suggesting a possible impairment of Cys conversion into GSH firstly controlled by glutamate-cysteine ligase (GCL). Oliveira *et al.* demonstrated, in fact, that the −129 C/T polymorphism in the promoter region of the *GCLC* gene encoding for the catalytic subunit of the GCL, was independently associated with NASH [29]. It has been reported, that this polymorphism may cause a 50%–60% decrease in the promoter activity of the *GCLC* gene in response to hydrogen peroxide in human endothelial cells [30]. Further investigation are required to understand if the unchanged levels of total GSH in NASH children may be imputable to a genetic predisposition such as the presence of the −129 C/T polymorphism of *GCLC* gene. In the case of GSH synthesis deregulation we expect an accumulation of Cys, but it does not occur because it should be recalled that in healthy individuals, depending on the need of the cell, some Cys is also generally incorporated into protein and some is degraded into sulfate and taurine [31]. Therefore, defective enzymes that catalyze these reactions could provide and additional explanation of Cys accumulation in children with NAFLD, but the mechanisms at the base of a potential “bad role” of excessive plasma levels of Cys remains to be elucidated.

An additional explanation of Hcy and Cys level reduction in plasma of patients with NAFLD could be its increased remethylation to methionine and consequent increase of methyl groups available for processes such as DNA methylation. Although plasma methionine levels were not measured in our cohort of patient, representing a possible limit of the present study, the excess of methyl donors groups is inconsistent because several authors have previously reported that methyl-depleted diets may promote NASH, while replenishing methyl stores prevent NAFLD progression in experimental models [32,33]. Furthermore, Murphy *et al.* found that human livers with advanced NAFLD are generally hypomethylated relative to those with mild NAFLD [34].

Histological assessment of liver damage remains crucial to confirm diagnosis of NASH that, in children, is characterized by several hepatic features such as portal and lobular inflammation, ballooning and eventually fibrosis [5]. Some experimental studies demonstrated that hyperhomocysteinemia may be involved in the liver injury occurring in NAFLD pathogenesis [20,22,23]. Werstuck *et al.* found that hyperhomocysteinemia induced in cultured human cells and murine models may cause endoplasmic reticulum (ER) stress, which in turn activates both the unfolded protein response and the sterol regulatory element–binding proteins (SREBPs) leading to a significant increase of intra-hepatic cholesterol and triglycerides levels suggesting a causal role of Hcy in steatosis [20]. Our results did not sustain these experimental findings because neither Hcy nor the other thiols correlate with the histological pattern of steatosis in our children cohort. On the contrary, Hcy and Cys plasma levels correlated with the presence of fibrosis according to a previous research study demonstrating that Hcy may promote the expression of metalloproteinases-1 (TIMP-1) in different liver-resident cells including hepatic stellate cells, hepatocytes and vascular smooth muscle cells [35]. Furthermore, Mattè *et al.* reported that chemically-induced chronic hyperhomocysteinemia in rats was able to induce not only increased oxidative stress and fibrosis but also the presence of inflammatory infiltrate in the liver [36]. Woo *et al.* reported that hyperhomocysteinemia increased superoxide anion production leading to lipid peroxidation in liver of rats, explaining many processes associated with Hcy-induced cell injury including apoptosis and inflammation in liver diseases [37]. In agreement, our results showed that plasma levels of Hcy significantly correlated with the presence of lobular/portal inflammation in children with NAFLD.

## 3. Experimental Section

### 3.1. Patients

Sixty-four children with biopsy-proven NAFLD and 64 age-, gender and body mass index (BMI) well-matched healthy subjects without evidence of fatty liver at ultrasound were included in this study. Patients were all enrolled after diagnosis the Hepato-Metabolic Unit of the Bambino Gesù Children’s Hospital between January 2012 and April 2013. Exclusion criteria for NAFLD and control subjects were alcohol consumption >20 g/day and use of medications that may induce liver disease or modify plasma levels of homocysteine (*i.e.*, folate, vitamin B12 and antibiotics). The absence of other causes of liver disease was also considered for NAFLD diagnosis. In particular, all patients with suspected NAFLD underwent complete laboratory investigation for: viral hepatitis (hepatitis B and C viral markers), sclerosing cholangitis, hemochromatosis, autoimmune hepatitis and primary biliary cirrhosis (non-organ-specific autoantibodies such as antinuclear antibody, anti-mitochondrial antibody, anti-smooth muscle antibody, and anti-liver/kidney microsomal antibody), and genetic diseases (α-1 antitripsin, ceruloplasmin). The study protocol was approved by the Ethical Committee of the Bambino Gesù Children’s Hospital and written informed consent was obtained from the parents of the children.

### 3.2. Anthropometric Data

Weight and height were measured after the subjects had removed their shoes and any heavy clothing using a standardized procedure. Overweight/obesity was defined by the presence of a BMI-calculated as the ratio of body weight to height [kg × (m^2^)^−1^] > 85th percentile.

### 3.3. Blood Assays

Blood samples were collected after an overnight fasting and immediately centrifuged to obtain plasma aliquots for the analysis of metabolic parameters and thiols. Alanine aminotransferase (ALT), aspartate aminotransferase (AST), γ-glutamyl-transferase (γGT), total triglycerides and cholesterol, glucose and insulin were measured by standard laboratory methods. The degree of insulin resistance was estimated with the homeostatic model assessment (HOMA) equation, as follows: [fasting insulin (μU/mL) × fasting glucose (mg/dL)/405] [38].

### 3.4. Plasma Thiols Determinations

Cysteine (Cys), total homocysteine (Hcy), cysteinylglycine (CysGly) and glutathione (GSH) levels were determined by using the derivatization and chromatography procedures performed as previously reported [16]. Briefly, 30 µL of 4 mol/L NaBH_4_, 20 µL of 2 mmol/L EDTA/DTT, 10 µL of 1-octanol and 20 µL of 1.8 mol/L HCl were placed in the derivatization vial containing 30 µL of sample. After the mixture was incubated for 3 min, 100 µL of 1.5 mol/L *N*-ethylmorpholine buffer, pH 8.0, 400 µL of distilled water, and 20 µL of 25 mmol/L bromobimane were added. After additional 3 min incubation, 40 µL of acetic acid was added, and 20 µL of this mixture was injected into a 150 mm × 4.6 mm Hypersil-ODS column (Thermo Fisher Scientific, Bellefonte, PA, USA) equilibrated with 30 mmol/L ammonium nitrate and 40 mmol/L ammonium formate buffer, pH 3.6 (A). *S*-Bimane adducts was eluted from the column in 6 min with a gradient of acetonitrile (B) (0–4 min, 0%–30% B; 4–5 min, 30%–100% B; 5–6 min, 100% B), at a flow rate of 1.5 mL/min. The HPLC system, with sample processor and solvent delivery system, was an Agilent Technologies 1100 equipped with fluorescence detector G1321A operating at an excitation wavelength of 390 nm and an emission wavelength of 478 nm; the data obtained were analyzed with the Agilent ChemStation^®^ software for Windows NT (Agilent Tecnologies, Waldbronn, Germany).

### 3.5. Liver Histology

Echo-guided liver biopsies were fixed in 10% buffered formalin and interpreted by an experienced pathologist (RDV) according to criteria proposed by NAFLD Clinical Research Network [39]. According to these guidelines: steatosis was graded 0–3 (0 = <5% steatosis, 1 = 5%–33%, 2 = 33%–66% and grade 3 = >66%); lobular inflammation was scored based on the number of inflammatory foci per 200× per field (0 = no inflammatory foci, 1 = <2 foci; 2 = 2–4 foci and 3 = >4 foci); ballooning was graded 0–2 (0 = none; 1 = few balloon cells present and 2 = prominent ballooning); fibrosis was staged 0–4 as follows: 0 = no fibrosis; 1 = periportal or perisinusoidal; 1A = mild, Zone 3, perisinusoidal; 1B = moderate, Zone 3, perisinusoidal; 1C = portal/periportal; 2 = perisinusoidal and portal/periportal; 3 = bridging fibrosis and 4 = cirrhosis.

### 3.6. Statistical Analysis

All statistical analyses were performed with software package SPSS (Ver. 12.0.2 for Windows, SPSS Inc., Chicago, IL, USA). Descriptive statistics for continuous variables were provided as the mean ± standard deviation (SD). Kolmogorov-Smirnov tests for normality of continuous data were performed. Categorical variables were expressed as number or percentage. Continuous variables were examined by a two-tailed Student’s *t* test. Correlations were calculated as Spearman correlation coefficients. Logistic regression models (with backward conditional method) were used, with adjustment for age and sex, to estimate OR expressed with a 95% confidence interval (CI). *p* < 0.05 was considered statistically significant.

## 4. Conclusions

In conclusion, our study demonstrates for the first time that Hcy and Cys plasma levels were significantly higher in NAFLD children than in controls making these thiols potential biomarkers of disease in a pediatric setting even if these results should be replicated in larger series. Furthermore, we found that Hcy and Cys plasma levels tend to increase in parallel with the severity of disease in terms of NAFLD-related fibrosis in children. However, further studies are required in order to characterize the potential thiols-driven pro-fibrogenic mechanism.
